# Lung Cancer Diagnosis Rates in Early Detection Programs in the Mississippi Delta

**DOI:** 10.1001/jamanetworkopen.2026.3171

**Published:** 2026-04-13

**Authors:** Wei Liao, Simon Tye, Jordan Goss, Carrie Fehnel, Meredith Ray, Raymond U. Osarogiagbon

**Affiliations:** 1 Thoracic Oncology Research Group, Multidisciplinary Thoracic Oncology Program, Baptist Cancer Center, Memphis, Tennessee; 2 Division of Epidemiology, Biostatistics, and Environmental Health, School of Public Health, University of Memphis, Memphis, Tennessee

## Abstract

**Importance:**

Estimates of the impact of lung cancer screening (LCS) largely rely on the 3.97% lung cancer diagnosis rate (LCDR) from the National Lung Screening Trial.

**Objective:**

To estimate the LCDR in clinical LCS and incidental pulmonary nodule (IPN) programs.

**Design, Setting, and Participants:**

This prospective cohort study of a large regional community health care system in the Mississippi Delta included patients who had at least 1 low-dose computed tomography (CT) scan for lung cancer or any CT scan finding an IPN diameter of 30 mm or less and no prior history of lung cancer between January 1, 2015, and June 30, 2024.

**Exposure:**

Patient surveillance in LCS or IPN programs.

**Main Outcomes and Measures:**

The primary outcome was aggregate LCDRs. Secondary outcomes were relative LCDRs stratified by baseline Lung CT Screening Reporting and Data System (Lung-RADS) score (LCS cohort) or nodule size (IPN cohort). Adjusted hazard ratios (AHRs) were estimated using a Cox proportional hazards model adjusted for age, sex, race, insurance, rurality, comorbidities, personal history of non–lung cancer, and family history of lung cancer.

**Results:**

Among 40 612 patients, 15 754 were enrolled in the LCS cohort (median [IQR] age, 65 [59-69] years; 7990 male [50.7%]) and 24 858 were enrolled in the IPN cohort (median [IQR] age, 64 [52-74] years; 13 919 female [56.0%]). Among patients in the LCS cohort, 13 517 (85.8%), 994 (6.3%), 559 (3.5%), 284 (1.8%), and 179 (1.1%) had Lung-RADS scores of 1 or 2, 3, 4A, 4B, and 4X, respectively. Among patients in the IPN cohort, 9204 (37.0%) had a baseline nodule diameter of less than 6 mm, 12 872 (51.8%) had a nodule of 6 to 15 mm, 1570 (6.3%) had a nodule of greater than 15 to 20 mm, and 1212 (4.9%) had a nodule of greater than 20 to 30 mm. The cumulative LCDR at 36 months was 3.8% (95% CI, 3.4%-4.1%) and 4.3% (95% CI, 4.1%-4.6%), with a median follow-up of 546 days (IQR, 237-996 days) and 647 days (IQR, 248-1279 days), in the LCS and IPN cohorts, respectively. With Lung-RADS 1 to 2 as the reference, AHRs were 3.38 (95% CI, 2.48-4.62), 7.41 (95% CI, 5.49-49.10), 25.46 (95% CI, 19.36-33.47), and 107.22 (95% CI, 82.76-138.19) for Lung-RADS 3, 4A, 4B, and 4X, respectively, and 0.59 (95% CI, 0.44-0.79), 2.66 (95% CI, 2.23-3.18), 9.93 (95% CI, 8.06-12.22), and 15.65 (95% CI, 12.67-19.32) for nodules less than 6 mm, 6 to 15 mm, greater than 15 to 20 mm, and greater than 20 to 30 mm in the IPN cohort. Five-year overall survival was 58% (95% CI, 52%-65%) and 46% (95% CI, 43%-50%) in the LCS vs IPN cohorts.

**Conclusions and Relevance:**

In this cohort study of LCS and IPN program patients, approximately 4% were diagnosed with lung cancer over less than half the duration of the NLST cohort, suggesting a greater population-level LCDR through these screening programs than estimated by clinical trial data.

## Key Points

**Question:**

What is the cumulative lung cancer diagnosis rate (LCDR) among patients enrolled in lung cancer screening (LCS) and incidental pulmonary nodule (IPN) programs in the Mississippi Delta?

**Findings:**

In this cohort study of 40 612 patients, cumulative LCDRs at 36 months were 3.8% and 4.3% in the LCS and IPN programs, respectively. The LCDR was similar to or exceeded the 3.97% reported in the National Lung Screening Trial after a median follow-up of 6.5 years.

**Meaning:**

These findings suggest that LCS and IPN programs may have greater outcomes associated with LCDRs than projected from clinical trial data.

## Teaser

This cohort study estimates the cumulative lung cancer diagnosis rate among patients enrolled in lung cancer screening and incidental pulmonary nodule programs in the Mississippi Delta.

## Introduction

The National Lung Screening Trial (NLST) demonstrated that lung cancer screening (LCS) by low-dose computed tomography (LDCT) reduced lung cancer mortality by 20% and all-cause mortality by 8%.^1^ Compared with the chest radiography cohort, the LDCT cohort had earlier stage disease, increased use of curative-intent treatment, and improved aggregate lung cancer survival.^2,3^ The NLST results, which were corroborated by the Dutch-Belgian trial NELSON and a meta-analysis,^4,5^ led directly to US Preventive Services Task Force (USPSTF) recommendations supporting lung cancer screening^6,7^ and a series of Medicare Coverage Decisions that established LCS as a covered health care benefit.^8,9^

However, because of healthy volunteers and a low proportion of Black participants, these trial results might not fully reflect lung cancer risk in diverse populations.^10,11^ Guideline-concordant treatment of patients with incidentally detected pulmonary nodules has also been associated with a shift to early-stage diagnosis, increased use of curative-intent treatment, and improved survival.^12^ Incidental pulmonary nodule (IPN) programs enroll diverse patients who often are ineligible for LCS by current screening criteria,^13^ including those with lung cancer risk not typically associated with smoking.^14,15^

We estimated cumulative lung cancer diagnosis rates (LCDRs) among participants in large community-based LCS and IPN programs in a regional US population with high lung cancer incidence and mortality rates, the Mississippi Delta.^16,17^ We also examined the characteristics and outcomes of lung cancer in both cohorts.

## Methods

This prospective cohort study was approved by the Institutional Review Board of Baptist Memorial Healthcare Corporation (BMHCC) with a waiver of informed consent as the study was deemed low risk to human participants. The study followed the Strengthening the Reporting of Observational Studies in Epidemiology (STROBE) reporting guideline for cohort studies.^19^

The cohort included patients enrolled in the LCS and IPN programs at BMHCC. This faith-based, not-for-profit community health care system serves a socioeconomically diverse population across more than 125 counties in Alabama, Arkansas, Mississippi, Missouri, Kentucky, and Tennessee, which have some of the highest US per-capita lung cancer incidence and mortality rates.^16,17^ Approximately 44% of the service area is made up of low-income and low–socially resourced Delta Regional Authority counties.^18^

### Detecting Early Lung Cancer in the Mississippi Delta Project

From 2015 onward, data on all patients enrolled in BMHCC’s LCS and IPN programs were collected in a Research Electronic Data Capture database (Vanderbilt University). Patients who underwent LDCT screening by contemporaneous USPSTF or Medicare eligibility criteria were identified using Healthcare Common Procedure Coding System code G0297 through January 1, 2021, and *Current Procedural Terminology* code 71271 from January 1, 2021, through June 30, 2024.^12^ In the IPN program, patients whose radiology report included a prespecified standard phrase indicating the need for further evaluation of an intrathoracic lesion were automatically identified by the electronic health record (EHR) (Epic; Epic Systems). Trained navigators used Fleischner Society guidelines to triage patients into care pathways.^12,14^

Trained data managers (including J.G.) abstracted data from the EHR into the prospective Detecting Early Lung Cancer in the Mississippi Delta database using standard operating procedures, including a data dictionary and audits. Race was self-reported and included African American or Black, White, or other (American Indian or Alaska Native, Asian, Native Hawaiian or Pacific Islander, and unknown). We combined individuals identifying as other than African American or Black and White because they were relatively few in number; race was included because of its association with lung cancer risk and variable access to LCS. Neither patients nor their clinicians were directly contacted by the research team. Vital statistics were obtained from the EHR and at prespecified biannual intervals, from the institutional tumor registry.

### Cohort Selection

This analysis included data from January 1, 2015, to June 30, 2024, with follow-up until January 1, 2025. We categorized the LCS cohort by the reported baseline American College of Radiology’s Lung CT Screening Reporting and Data System (Lung-RADS) score, as follows: 1 (negative) or 2 (benign nodule) recommended for 12-month LDCT; 3 (probably benign), recommended for 6-month LDCT; 4A (suspicious), recommended for 3-month LDCT or positron emission tomography (PET)–CT scan; 4B (very suspicious), recommended for diagnostic CT, PET-CT, or biopsy; and 4X (very suspicious and additional features), recommended for diagnostic CT, PET-CT, or biopsy.^20^ We categorized the IPN cohort by baseline lesion size (<6 mm, 6 to 15 mm, >15 to 20 mm, and >20 to 30 mm).^21^

**Figure 1.**
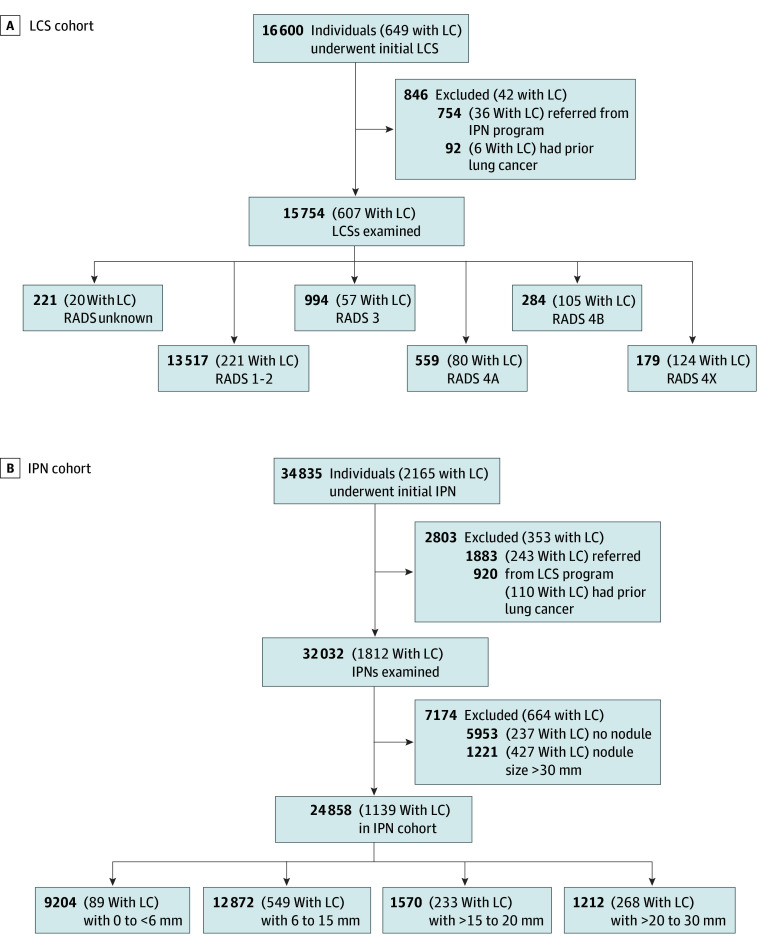
Flow Diagram of the Derivation of the Lung Cancer Screening (LCS) and Incidental Pulmonary Nodule (IPN) Cohorts The initial cohorts are shown, with exclusions applied sequentially, and the remaining patients stratified by Lung Computed Tomography Imaging Reporting and Data System (Lung-RADS) score and nodule size. Exclusion criteria included referral from another early-detection program, prior history of lung cancer (LC), absence of pulmonary nodules, or nodules larger than 30 mm.

We excluded all patients referred from a different early detection program, those with a prior history of lung cancer, or those with IPNs larger than 30 mm (Figure 1). Prior lung cancer was identified through the EHR. We categorized patients using their baseline radiologic findings, regardless of subsequent Lung-RADS score or nodule size.

### Variables of Interest

We compared patient demographic and socioeconomic characteristics (age, sex, race, insurance, rurality of place of residence), smoking history (smoking status [never, active, former], intensity [pack-years], quit duration), comorbid conditions (eg, chronic obstructive pulmonary disease [COPD]), and nodule characteristics (size, location, attenuation, edge characteristics, presence of calcification or cavitation). We also captured tumor histologic type; size; stage; treatment; and vital status, including date of death.

### Statistical Analysis

We summarized demographic, clinical, and lung cancer characteristics using descriptive statistics, including frequency (percentage), mean (SD), and median (IQR). To compare characteristics, we used χ^2^ tests (Fisher exact test if cell counts were expectedly small) and Cramer *V* for effect sizes for categorical variables and Wilcoxon rank sum test and rank biserial correlation for effect sizes for continuous variables. We reported missingness for each variable, used all available data for descriptive comparisons, and applied complete-case analysis to multivariable regressions.

We modeled time to diagnosis by cohort via the cumulative incidence function using a Fine-Gray subdistribution hazards model. This approach accounted for competing risks (eg, death, loss to follow-up). Time to diagnosis was measured from time 0 (the date of the first LDCT screening or initial nodule detection scan) until the date of lung cancer diagnosis (event of interest), non–lung cancer cause of death (competing interest), and last contact (loss to follow-up) or January 1, 2025 (censored). We provide cumulative incidence diagnosis rates with 95% CIs for up to 3 years in 6-month increments (0-6 months, 0-12 months, etc). We also calculated the incidence rates by person-years to account for varying follow-up times among cohorts.

Among patients diagnosed with lung cancer, we estimated overall survival for each cohort from time of diagnosis to death of any cause, with censoring at last contact or on January 1, 2025, using the Kaplan-Meier method and compared survival using the log-rank test. Adjusted hazard ratios (AHRs) were estimated using a complete-case Cox proportional hazards model, with cohort as the primary estimator and adjustment for age, sex, race, insurance, rurality, comorbidities, personal history of non–lung cancer, and family history of lung cancer. This analysis assumed noninformative censoring. Time-to-event analyses included all patients with observed event or censoring data.

We conducted subset analyses within cohorts. We compared characteristics and LCDRs across Lung-RADS categories (1-2, 3, 4A, 4B, and 4X) in the LCS cohort and initial nodule size (<6 mm, 6 to 15 mm, >15 to 20 mm, and >20 to 30 mm) in the IPN cohort. We also modeled time to diagnosis as a function of cohort, Lung-RADS, or nodule size categories using Cox proportional hazard models. Hazard ratios were estimated unadjusted and adjusted for age, sex, race, insurance, rurality, comorbidities, personal history of non–lung cancer, and family history of lung cancer. Time to diagnosis was defined from time 0 to diagnosis and censored at time of death or last contact. Analyses were performed using R, version 4.1.1 (R Foundation for Statistical Computing), and tests were 2-sided with a significance threshold of *P* < .05.

## Results

### Cohort Characteristics

Among 40 612 patients who met the inclusion criteria (Figure 1), 15 754 (38.8%) were enrolled in the LCS cohort (median [IQR] age, 65 [59-69] years; 7763 female [49.3%] and 7990 male [50.7%]; 3313 identifying as African American or Black [21.0%], 12 040 as White [76.4%], and 401 as other [2.6%] race; 6100 living rurally [38.7%]; 231 uninsured [1.5%]), and 24 858 (61.2%) participated in the IPN program (median [IQR] age, 64 [52-74] years; 13 919 female [56.0%] and 10 936 male [44.0%]; 7107 identifying as African American or Black [28.6%], 16 738 as White [67.3%], and 1013 as other [4.1%] race; 6340 living rurally [25.5%]; 1813 uninsured [7.3%]) (Table 1). Among patients in the LCS cohort, 13 517 (85.8%), 994 (6.3%), 559 (3.5%), 284 (1.8%), and 179 (1.1%) had Lung-RADS 1 or 2, 3, 4A, 4B, and 4X, respectively. Among patients in the IPN cohort, 9204 (37.0%), 12 872 (51.8%), 1570 (6.3%), and 1212 (4.9%) had a baseline nodule diameter of less than 6 mm, 6 to 15 mm, greater than 15 to 20 mm, and greater than 20 to 30 mm, respectively. eFigure 1 in Supplement 1 shows the percentage of patients who were and were not diagnosed with lung cancer in the LCS and IPN cohorts.

**Table 1.** Cohort Characteristics of DELUGE Enrollees

Characteristic	Patients, No. (%)			
	LCS (n = 15 754)	IPN (n = 24 858)	LCS lung cancer (n = 607)	IPN lung cancer (n = 1139)
**Demographic and clinical **				
Lung cancer	607 (3.9)	1139 (4.6)	607 (100)	1139 (100)
Age at enrollment, y				
Mean (SD)	64.4 (6.9)	62.2 (15.2)	67.7 (6.1)	69.6 (9.6)
Median (IQR) [range]	65 (59-69) [26-94]	64 (52-74) [10-107]	68 (64-72) [50-87]	70 (63-76) [37-100]
Sex				
Female	7990 (50.7)	13 919 (56.0)	312 (51.4)	604 (53.0)
Male	7763 (49.3)	10 936 (44.0)	295 (48.6)	534 (46.9)
Missing	1 (<0.1)	3 (<0.1)	0	1 (0.1)
Race				
African American or Black	3313 (21.0)	7107 (28.6)	109 (18.0)	262 (23.0)
White	12 040 (76.4)	16 738 (67.3)	490 (80.7)	854 (75.0)
Other^a^	401 (2.6)	1013 (4.1)	8 (1.3)	23 (2.0)
Insurance				
Medicare	8764 (55.6)	11 941 (48.0)	422 (69.5)	791 (69.5)
Medicaid	583 (3.7)	776 (3.1)	19 (3.1)	25 (2.2)
Commercial	6176 (39.2)	10 328 (41.6)	161 (26.5)	280 (24.6)
Self-insured or unknown	231 (1.5)	1813 (7.3)	5 (0.8)	43 (3.8)
RUCA code				
Metro area	9652 (61.3)	18 492 (74.4)	394 (64.9)	842 (73.9)
Rural area	6100 (38.7)	6340 (25.5)	213 (35.1)	297 (26.1)
Missing	2 (<0.1)	26 (0.1)	0	0
Smoking status				
Active	10 758 (68.3)	6774 (27.3)	439 (72.3)	567 (49.8)
Former	4918 (31.2)	6899 (27.8)	168 (27.7)	446 (39.2)
Never	49 (0.3)	10 253 (41.3)	0	118 (10.4)
Missing	29 (0.2)	932 (3.8)	0	8 (0.7)
Pack-years				
Sample size, No.	15 676	13 673	607	1013
0 to <10	246 (1.6)	4198 (30.7)	4 (0.7)	113 (11.2)
10 to <20	542 (3.5)	1247 (9.1)	16 (2.6)	86 (8.5)
20 to <30	1756 (11.2)	1163 (8.5)	50 (8.2)	112 (11.1)
30 to <40	3086 (19.7)	951 (7.0)	60 (9.9)	84 (8.3)
≥40	8620 (55.0)	2826 (20.7)	449 (74.0)	535 (52.8)
Missing	1426 (9.1)	3288 (24.1)	28 (4.6)	83 (8.2)
Quit duration, y				
Sample size, No.	4918	6899	168	446
0-15	4263 (86.7)	2092 (30.3)	152 (90.5)	191 (42.8)
16-25	335 (6.8)	911 (13.2)	10 (6.0)	76 (17.0)
≥25	216 (4.4)	1875 (27.2)	5 (3.0)	137 (30.7)
Missing	104 (2.1)	2021 (29.3)	1 (0.6)	42 (9.4)
USPSTF 2021				
Eligible	12 933 (82.1)	3192 (12.8)	542 (89.3)	548 (48.1)
Ineligible	2821 (17.9)	21 666 (87.2)	65 (10.7)	591 (51.9)
COPD				
No	9868 (62.6)	20 563 (82.7)	282 (46.5)	643 (56.5)
Yes	5886 (37.4)	4295 (17.3)	325 (53.5)	496 (43.6)
Comorbidities				
0	4549 (28.9)	9301 (37.4)	106 (17.5)	231 (20.3)
1	7044 (44.7)	9336 (37.6)	300 (49.4)	510 (44.8)
2	4161 (26.4)	6221 (25.0)	201 (33.1)	398 (34.9)
Prior history of non–lung cancer				
No	12 960 (82.3)	19 887 (80.0)	456 (75.1)	841 (73.8)
Yes	2794 (17.7)	4971 (20.0)	151 (24.9)	298 (26.2)
Family history of cancer				
No	4198 (26.7)	8217 (33.1)	160 (26.4)	324 (28.5)
Yes	7887 (50.1)	9556 (38.4)	358 (59.0)	654 (57.4)
Unknown	3669 (23.3)	7085 (28.5)	89 (14.7)	161 (14.1)
Family history of lung cancer				
No	6109 (38.8)	7673 (30.9)	238 (39.2)	429 (37.7)
Yes	1778 (11.3)	1883 (7.6)	120 (19.8)	225 (19.8)
**Lesion characteristics**				
No. of lesions				
0	7066 (44.9)	0	116 (19.1)	0
1	3331 (21.1)	16 715 (67.2)	188 (31.0)	788 (69.2)
>1	5353 (34.0)	8143 (32.8)	303 (49.9)	351 (30.8)
Missing	4 (<0.1)	0	0	0
Size of largest, mm				
Mean (SD)	5.9 (10.7)	8.5 (5.6)	18.3 (17.7)	15.2 (7.1)
Median (IQR) [range]	4 (2-6) [1-707]	7 (5-10) [1-30]	13 (6-24) [1-120]	15 (9.55-20) [1.85-30]
Location				
Sample size, No.	8684	24 858	491	1139
RUL	2832 (32.6)	5604 (22.5)	162 (33.0)	348 (30.6)
RML	847 (9.8)	3043 (12.2)	28 (5.7)	85 (7.5)
RLL	1570 (18.1)	6214 (25.0)	99 (20.2)	219 (19.2)
LUL	1859 (21.4)	4183 (16.8)	124 (25.3)	313 (27.5)
LLL	1344 (15.5)	5018 (20.2)	67 (13.7)	154 (13.5)
Mediastinum	36 (0.4)	166 (0.7)	8 (1.6)	4 (0.4)
Unknown or NOS	196 (2.3)	630 (2.5)	3 (0.6)	16 (1.4)
Edge characteristics				
Sample size, No.	8684	24 858	491	1139
Smooth	4 (0.1)	39 (0.2)	0	1 (0.1)
Scalloped	0	5 (<0.1)	0	1 (0.1)
Spiculated	319 (3.7)	1040 (4.2)	123 (25.1)	247 (21.7)
Not reported	8165 (94.0)	23 412 (94.2)	340 (69.3)	844 (74.1)
Lobulated	69 (0.8)	177 (0.7)	22 (4.5)	39 (3.4)
Well circumscribed	101 (1.2)	142 (0.6)	5 (1.0)	6 (0.5)
Well marginated	26 (0.3)	43 (0.2)	1 (0.2)	1 (0.1)
Density				
Sample size, No.	8684	24 858	491	1139
Ground glass opacity	536 (6.2)	1487 (6.0)	29 (5.9)	82 (7.2)
Solid	807 (9.3)	1810 (7.3)	64 (13.0)	76 (6.7)
Part-solid	187 (2.2)	370 (1.5)	32 (6.5)	51 (4.5)
Not reported	7151 (82.4)	21 180 (85.2)	366 (74.5)	929 (81.6)
Honeycomb	3 (<0.1)	11 (<0.1)	0	1 (0.1)

Abbreviations: COPD, chronic obstructive pulmonary disease; DELUGE, Detecting Early Lung Cancer; IPN, incidental pulmonary nodule; LCS, lung cancer screening; LLL, lower left lobe; LUL, left upper lobe; NOS, not otherwise specified; RLL, right lower lobe; RML, right middle lobe; RUCA, Rural-Urban Commuting Area; RUL, right upper lobe; USPSTF, US Preventive Services Task Force.

^a^ Included American Indian or Alaska Native, Asian, Native Hawaiian or Pacific Islander, or unknown.

### Lung Cancer Risk Factors and Pulmonary Nodule Radiologic Characteristics

In the LCS cohort, 10 758 patients (68.3%) smoked compared with 6774 (27.3%) in the IPN cohort (Cramer *V* = 0.50; *P* < .001) (Table 1). In the IPN cohort, 10 253 (41.3%) never smoked; 6899 (27.8%) quit smoking; and among those who smoked, 5445 of 13 673 (39.8%) smoked less than 20 pack-years. Among patients who quit smoking in the IPN cohort, 2786 (40.4%) had quit for more than 15 years. Only 3786 patients (15.2%) in the IPN cohort would have met USPSTF 2021 smoking history criteria, and 3192 (12.8%) were eligible for LCS. In the LCS cohort, COPD was more common compared with the IPN cohort (5886 [37.4%] vs 4295 [17.3%]; Cramer *V* = 0.23; *P* < .001), as were a family history of cancer (7887 [50.1%] vs 9556 [38.4%]; Cramer *V* = 0.11; *P* < .001) and lung cancer (1778 [11.3%] vs 1883 [7.6%]; Cramer *V* = 0.04; *P* < .001). The LCS cohort compared with the IPN cohort had smaller nodules (median [IQR], 4 [2-6] mm vs 7 [5-10] mm; *r* = 0.35; *P* < .001) and was less likely to have a solitary nodule (3331 [21.1%] vs 16 715 [67.2%]; Cramer *V* = 0.61; *P* < .001).

### Characteristics of Patients With Lung Cancer and Their Baseline Nodules

Overall, 607 (3.9%) and 1139 (4.6%) patients were diagnosed with lung cancer in the LCS and IPN cohorts, respectively (Table 1). Among these patients, the LCS cohort was younger than the IPN cohort (median [IQR] age, 68 [64-72] years vs 70 [63-76] years; *r* = 0.12; *P* < .001) and less often of African American or Black race (109 [8.0%] vs 262 [23.0%]; Cramer *V* = 0.06; *P* = .01). Among patients in the IPN cohort who were diagnosed with lung cancer, only 548 (48.1%) met USPSTF 2021 smoking history criteria for LCS eligibility. The LCS cohort compared with the IPN cohort had proportionately more people who currently smoked and fewer who had quit smoking (439 [72.3%] vs 567 [49.8%]; 168 [27.7%] vs 446 [39.2%]; Cramer *V* = 0.25; *P* < .001); 118 patients (10.4%) in the IPN cohort had never smoked. Although there was no difference in the distribution of comorbidities (Cramer *V* = 0.15; *P* = .15), COPD was more prevalent in the LCS cohort compared with the IPN cohort (325 [53.5%] vs 496 [43.6%]; Cramer *V* = 0.10; *P* < .001). The nodule size was similar between the LCS and IPN cohorts (median [IQR], 13 [6-24] mm vs 15 [10-20] mm, respectively; *r* = 0.05, *P* = .07). The LCS cohort compared with the IPN cohort more often had multiple nodules (303 [49.9%] vs 351 [30.8%]; Cramer *V* = 0.45; *P* < .001).

### Lung Cancer Characteristics and Treatment

The distribution of histologic type differed between the LCS and IPN cohorts (Cramer *V* = 0.16; *P* < .001) (eTable 1 in Supplement 1) mostly because of differences in the proportions with adenocarcinoma (239 [39.4%] vs 612 [53.7%]) and squamous cell carcinoma (212 [34.9%] vs 254 [22.3%]). The number of patients diagnosed through the IPN program greatly exceeded LCS enrollees across histologic types (eTable 1 in Supplement 1). Although tumor size did not differ in the LCS cohort compared with the IPN cohort (median [IQR], 2.2 [1.4-3.4] cm vs 2.1 [1.5-3.0] cm; *r* = .02; *P* = .48), the distribution of clinical stage differed (Cramer *V* = 0.08; *P* = .03). Similar proportions of patients in the LCS and IPN cohorts were diagnosed with stage I (330 [54.4%] vs 594 [52.1%], respectively) and fewer with stage IV (105 [17.3%] vs 236 [20.7%], respectively). Nevertheless, the IPN cohort had more patients across stages. While the distribution of treatment modalities did not differ between cohorts (Cramer *V* = 0.07; *P* = .30), 315 (51.9%) in the LCS cohort vs 530 (46.5%) in the IPN cohort received definitive curative-intent local treatment (defined as surgery or stereotactic body radiation therapy).

### Lung Cancer Diagnosis Rates

**Figure 2.**
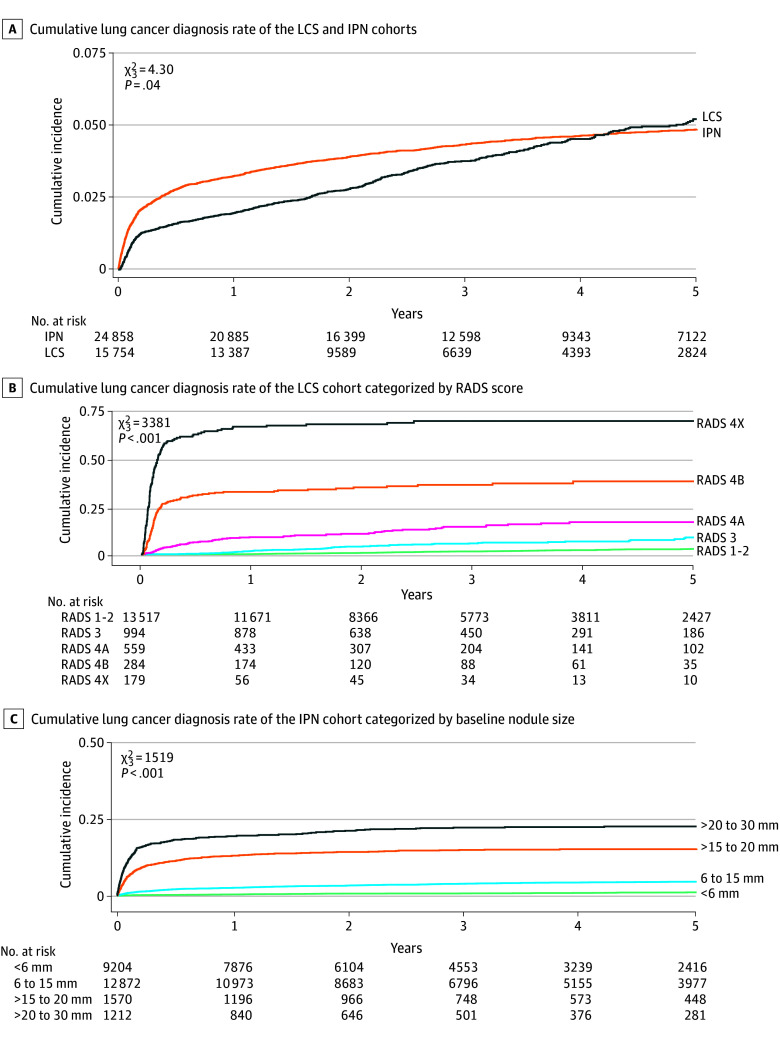
Kaplan-Meier Plot of Cumulative Lung Cancer Diagnosis Rates for the Lung Cancer Screening (LCS) and Incidental Pulmonary Nodule (IPN) Cohorts Differences in cumulative incidence were assessed using the Fine-Gray test. RADS indicates Lung Computed Tomography Imaging Reporting and Data System.

The cumulative LCDR increased in 6-month increments (Table 2), peaking at 3.8% (95% CI, 3.4%-4.1%) and 4.3% (95% CI, 4.1%-4.6%) in the LCS and IPN cohorts, respectively, at 36 months (Figure 2). The cumulative incidence of lung cancer diagnosis was 13.0 per 1000 person-years in the LCS cohort (5.4, 18.8, 53.0, 189.1, and 684.3 per 1000 person-years for Lung-RADS scores 1-2, 3, 4A, 4B, and 4X, respectively) and 13.1 per 1000 person-years in the IPN cohort (2.8, 11.7, 45.5, and 80.0 per 1000 person-years for nodules <6 mm, 6 to 15 mm, >15 to 20 mm, and >20 to 30 mm, respectively). The comparative LCDR rates were 1.6% (95% CI, 1.3%-1.8%), 5.8% (95% CI, 4.2%-7.6%), 14.4% (95% CI, 11.2%-17.9%), 36.3% (95% CI, 30.4%-42.2%), and 69.6% (95% CI, 62.0%-76.0%) for Lung-RADS scores 1 to 2, 3, 4A, 4B, and 4X, respectively, and 0.8% (95% CI, 0.6%-1.0%), 3.9% (95% CI, 3.6%-4.3%), 14.9% (95% CI, 13.1%-16.8%), and 22.2% (95% CI, 19.8%-24.6%) for nodules less than 6 mm, 6 to 15 mm, greater than 15 to 20 mm, and greater than 20 to 30 mm, respectively.

**Table 2.** Lung Cancer Diagnosis Rate of DELUGE Enrollees Stratified by Lung-RADS Scores or Nodule Size

Variable	LCS cohort						IPN cohort				
	Total	Lung-RADS 1-2	Lung-RADS 3	Lung-RADS 4A	Lung-RADS 4B	Lung-RADS 4X	Total	<6 mm	6 to 15 mm	>15 to 20 mm	>20 to 30 mm
Total No. of patients	15 754	13 517	994	559	284	179	24 858	9204	12 872	1570	1212
Subgroup characteristics											
No. of patients with lung cancer	607	221	57	80	105	124	1139	89	549	233	268
Cumulative incidence of lung cancer diagnosis, per 1000 person-years	13.0	5.4	18.8	53.0	189.1	684.3	13.1	2.8	11.7	45.5	80.0
**Cumulative incidence of lung cancer diagnosis, % (95% CI)**											
0-6 mo	1.6 (1.4-1.8)	0.1 (0.05-0.1)	0.3 (0.1-0.9)	6.3 (4.5-8.6)	30.8 (25.4-36.3)	62.6 (55.0-69.4)	2.8 (2.6-3.0)	0.3 (0.2-0.4)	2.1 (1.9-2.4)	11.3 (9.7-12.9)	18.2 (16.1-20.5)
0-12 mo	2.0 (1.8-2.2)	0.1 (0.1-0.2)	1.8 (1.1-2.8)	9.0 (6.8-11.6)	32.6 (27.2-38.2)	66.7 (59.1-73.2)	3.2 (3.0-3.5)	0.4 (0.3-0.6)	2.6 (2.3-2.8)	13 .0 (11.4-14.8)	19.4 (17.2-21.7)
0-18 mo	2.4 (2.2-2.6)	0.5 (0.4-0.6)	2.7 (1.8-3.9)	10.0 (7.6-12.7)	33.5 (28.0-39.1)	68.0 (60.4-74.4)	3.6 (3.4-3.9)	0.6 (0.4-0.8)	3.1 (2.8-3.4)	13.8 (12.1-15.5)	20.0 (17.7-22.3)
0-24 mo	2.8 (2.5-3.1)	0.8 (0.6-1.0)	4.1 (2.9-5.6)	10.7 (8.2-13.6)	35.0 (29.3-40.7)	68.0 (60.4-74.4)	3.9 (3.7-4.2)	0.7 (0.5-0.9)	3.3 (3.0-3.6)	14.2 (12.5-16.0)	21.2 (18.8-23.6)
0-30 mo	3.4 (3.1-3.7)	1.2 (1.0-1.5)	5.2 (3.8-6.9)	12.9 (10.0-16.2)	35.6 (29.9-41.4)	69.6 (62.0-76.0)	4.1 (3.9-4.4)	0.7 (0.6-0.9)	3.6 (3.3-3.9)	14.7 (13.0-16.5)	21.8 (19.4-24.2)
0-36 mo	3.9 (3.4-4.1)	1.6 (1.3-1.8)	5.8 (4.2-7.6)	14.4 (11.2-17.9)	36.3 (30.4-42.2)	69.6 (62.0-76.0)	4.3 (4.1-4.6)	0.8 (0.6-1.0)	3.9 (3.6-4.3)	14.9 (13.1-16.8)	22.2 (19.8-24.6)
**Unadjusted HR (95% CI)**											
No. with complete data	11 809	10 123	742	423	213	140	16 738	6110	8666	1107	855
No. with missing data	3945	3394	252	136	71	39	8120	3094	4206	463	357
Aggregate^a^	NA	1 [Reference]	3.60 (2.64-4.90)	8.13 (6.04-10.93)	29.34 (22.47-38.37)	113.17 (88.09-145.38)	NA	0.22 (0.17-0.28)	1 [Reference]	4.02 (3.39-4.76)	6.33 (5.37-7.46)
*P* value^b^	NA	NA	<.001	<.001	<.001	<.001	NA	<.001	NA	<.001	<.001
Aggregate^c^	NA	1 [Reference]	3.60 (2.64-4.90)	8.13 (6.04-10.93)	29.37 (22.47-38.37)	113.17 (88.09-145.38)	2.90 (2.48-3.40)	0.52 (0.39-0.69)	2.52 (2.12-3.00)	10.12 (8.28-12.37)	16.43 (13.50-19.98)
*P* value^b^	NA	NA	<.001	<.001	<.001	<.001	<.001	<.001	<.001	<.001	<.001
**AHR (95% CI)** ^d^											
Aggregate^a^	1 [Reference]	1 [Reference]	3.38 (2.48-4.62)	7.41 (5.49-49.10)	25.46 (19.36-33.47)	107.22 (82.76-138.19)	NA	0.23 (0.18-0.30)	1 [Reference]	3.87 (3.27-4.58)	5.80 (4.92-6.85)
*P* value^b^	NA	NA	<.001	<.001	<.001	<.001	NA	<.001	NA	<.001	<.001
Aggregate^c^	NA	1 [Reference]	3.38 (2.48-4.62)	7.41 (5.49-49.10)	25.46 (19.36-33.47)	107.22 (82.76-138.19)	3.10 (2.64-3.64)	0.59 (0.44-0.79)	2.66 (2.23-3.18)	9.93 (8.06-12.22)	15.65 (12.67-19.32)
*P* value^b^	NA	NA	<.001	<.001	<.001	<.001	<.001	<.001	<.001	<.001	<.001

Abbreviations: AHR, adjusted hazard ratio; DELUGE, Detecting Early Lung Cancer; HR, hazard ratio; IPN, incidental pulmonary nodule; LCS, lung cancer screening; Lung-RADS, Lung Computed Tomography Imaging and Reporting and Data System; NA, not applicable.

^a^ Shows HRs compared with the reference group (LCS Lung-RADS 1-2 for LCS cohort; 16-15 mm for the IPN cohort).

^b^ For comparing HRs, *P* values were derived from Wald tests within a Cox proportional hazards model, with patients censored due to death, loss to follow-up, or survival.

^c^ Shows additional contrasts between categories with the LCS Lung-RADS 1-2 group as the universal reference (ie, comparisons among groups in LCS and the IPN cohorts).

^d^ Adjusted for age, sex, race, insurance, rurality, comorbidities, personal history of non–lung cancer, and family history of lung cancer.

### Survival

**Figure 3.**
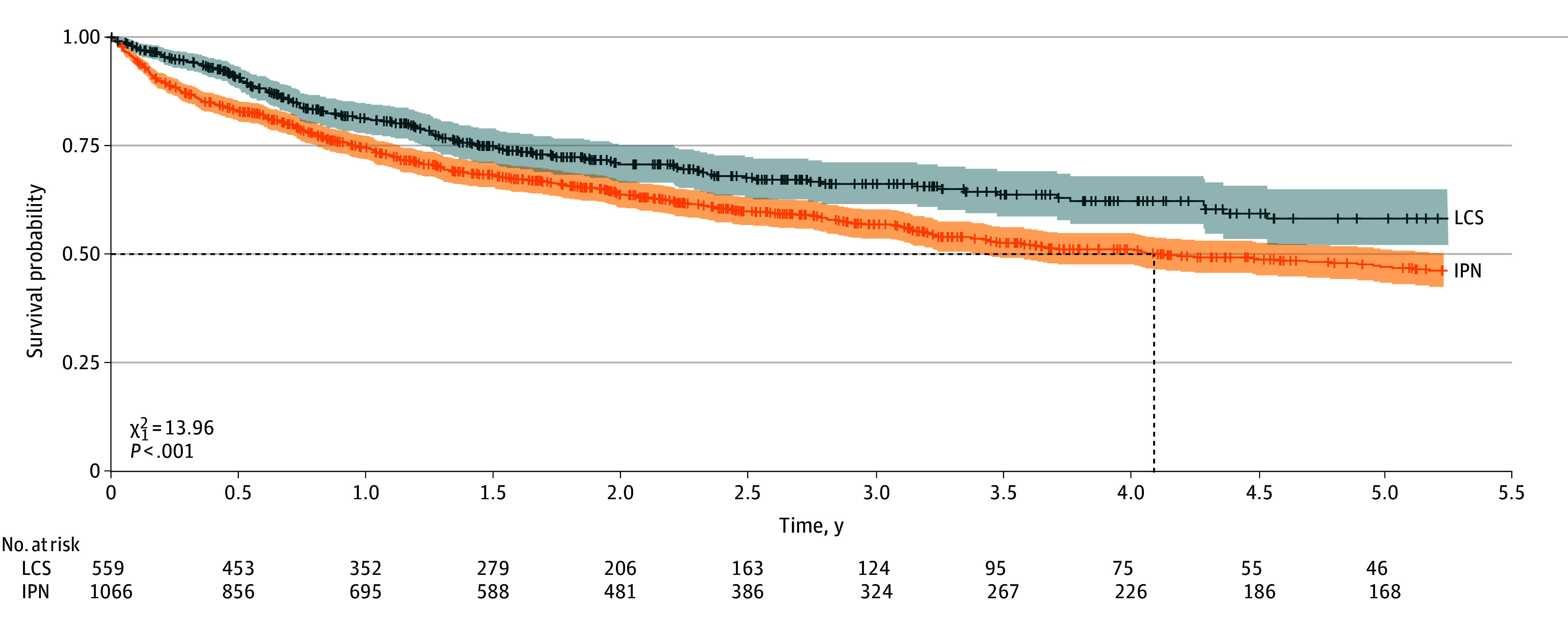
Survival Plot Comparing Patients Diagnosed With Lung Cancer in the Lung Cancer Screening (LCS) and Incidental Pulmonary Nodule (IPN) Cohorts The survival curves were compared using the log-rank test. Dashed horizontal line indicates the 50% survival probability and dashed vertical line, the median survival. Shading indicates the 95% CI and tic marks, censoring.

After a median duration of follow-up from diagnosis of 546 days (IQR, 237-996 days) in the LCS cohort and 647 days (248-1279 days) (*r* = .06; *P* = .01) in the IPN cohort, the median overall survival was not reached vs 4.09 years (95% CI, 3.42-5.30 years) (Figure 3; eTable 1 in Supplement 1). The aggregate 5-year overall survival was 58% (95% CI, 52%-65%) and 46% (95% CI, 43%-50%) in the respective cohorts (*P* < .001). Within-cohort overall survival estimates seemed similar across baseline Lung-RADS score or nodule size based on adjusted analyses (eFigure 2 and eTables 2 and 3 in Supplement 1).

### Adjusted Hazards for Lung Cancer Diagnosis

With LCS as reference, the HR and AHR for survival among patients with lung cancer were 2.90 (95% CI, 2.48-3.40; *P* < .001) and 3.10 (95% CI, 2.64-3.64; *P* < .001), respectively, for the IPN cohort. With Lung-RADS 1 or 2 as the reference, the AHR for lung cancer diagnosis was 3.38 (95% CI, 2.48-4.62), 7.41 (95% CI, 5.49-49.10), 25.45 (95% CI, 19.36-33.47), and 107.22 (95% CI, 82.76-138.19) for Lung-RADS 3, 4A, 4B, and 4X, respectively, in the LCS cohort (all *P* < .001) (Table 2; eFigure 3 in Supplement 1). With nodule sizes of 6 to 15 mm as the reference, AHR was 0.23 (95% CI, 0.18-0.30), 3.87 (95% CI, 3.27-4.58), and 5.80 (95% CI, 4.92-6.85) among patients in the IPN cohort with nodules less than 6 mm, greater than 15 to 20 mm, and greater than 20 to 30 mm, respectively (all *P* < .001). Using the Lung-RADS 1 or 2 cohort as the reference, the AHR was 0.59 (95% CI, 0.44-0.79), 2.66 (95% CI, 2.23-3.18), 9.93 (95% CI, 8.06-12.22), and 15.65 (95% CI, 12.67-19.32) for the IPN cohort with nodules less than 6 mm, 6 to 15 mm, greater than 15 to 20 mm, and greater than 20 to 30 mm, respectively (all *P* < .001). Complete-case analyses showed similar AHRs across Lung-RADS categories in the LCS cohort and nodule size categories in the IPN cohort (Table 2).

## Discussion

This community-based prospective cohort study of patients from the Mississippi Delta found that approximately 4% of patients in both the LCS and IPN programs were diagnosed with lung cancer, with respective 36-month cumulative diagnosis rates of 3.8% and 4.3% compared with the 3.97% rate after a median of 6.5 years in the NLST.^1^ The cumulative risk in both cohorts increased beyond 3 years, similar to the screened NLST cohort in which 6.4% were diagnosed after a median follow-up of 11.3 years.^22^

Lung cancer risk profiles differed between cohorts. The IPN cohort had less cigarette exposure (10.4% never smoked), fewer smoking-related comorbidities, and a broader age range. Only 548 of the 1139 patients (48.1%) diagnosed through the IPN program would have qualified for LCS using USPSTF 2021 criteria, and more racially minoritized patients were diagnosed through the IPN program. These findings highlight the critical complementary role of IPN programs, which capture at-risk populations missed by current guideline-based LCS.^23,24^ Personal and family history of cancer did not differ between cohorts.

Baseline Lung-RADS score and pulmonary nodule size were associated with cumulative LCDR,^21,25^ reaffirming their utility for triaging patients into care pathways.^20,25,26^ The distribution of baseline Lung-RADS scores in our LCS cohort (85.8%, 6.3%, 3.5%, 1.8% and 1.1% for Lung-RADS 1 or 2, 3, 4A, 4B, and 4X, respectively) was similar to the population prevalence reported by the American College of Radiology (84%, 9%, 4%, 2%, and <1%, respectively).^27^ The cumulative LCDR was higher in the IPN cohort during the first year, after which it increased more steeply in the LCS cohort, with the curves crossing at approximately 4.5 years. This finding emphasizes the need for adherence to LCS throughout the years of eligibility. While initial risk was greater in the IPN cohort, long-term surveillance remains important for all patients.^28^

There were proportionately more patients with squamous cell carcinoma and fewer with adenocarcinoma in the LCS cohort. The distribution of treatment modalities was similar, but clinical stage skewed slightly earlier in the LCS cohort, and a slightly greater proportion received definitive local curative-intent treatment (surgery and stereotactic body radiation). Nevertheless, substantially more patients were diagnosed at an early stage and received definitive local curative-intent treatment in the IPN cohort.

Our study has several implications. First, with less than half the duration of follow-up of the NLST, this diverse population showed a similar LCDR as reported in the NLST, suggesting that LCS may detect more cancer with a longer duration of follow-up and consistent with findings from a community-based screening cohort in England.^29^ If corroborated, these findings suggest that NLST data may underestimate the actual impact of LCS, highlighting the need for multilevel strategies to expand access to early detection. Second, current LCS eligibility criteria remain inadequate. The USPSTF 2021 criteria excluded 591 of the 1139 patients (51.9%) diagnosed with lung cancer in the IPN program, approximately the total number (607 persons) diagnosed through the LCS program. These criteria miss many patients who develop cancer,^23,24^ and the main eligibility barrier is the smoking history requirement.^14,30^ Our findings support modifying LCS eligibility criteria to capture a larger, more diverse population with heterogeneous risk factors.^11,31,32^ Third, maximal population-level benefit requires the implementation of LCS, IPN, and smoking cessation programs.^33^

### Limitations

This study had some limitations. It was limited by missing data, misclassification risk (eg, smoking history in the IPN cohort), a lack of causal attribution, and incomplete follow-up; only baseline Lung-RADS scores and nodule sizes were analyzed. Reliable information about other lung cancer risk factors (eg, radon, asbestos) were unavailable. Restricting the IPN cohort to 30 mm or smaller baseline nodule size may have underestimated the LCDR.

## Conclusions

This cohort study of patients participating in clinical LCS and IPN programs found that LCS may have greater population-level outcomes associated with LCDRs than suggested by the NLST (because the LCS cohort had less than half the duration of follow-up of the NLST cohort), and screening eligibility criteria missed many at-risk populations. Implementing both LCS and IPN programs enhanced the population-level outcomes associated with early detection, and the Lung-RADS scoring system and baseline nodule size remained robust cancer risk estimators. Extending early detection to all at-risk populations will require robust implementation science–based approaches to improve the deployment of evidence-based programs, along with novel strategies to enhance population selection, including biomarker testing^34^ and artificial intelligence.^35^

## Notes

Supplement 1. **eTable 1.** Cancer Characteristics of DELUGE Patients With Lung Cancer **eTable 2.** Survival Analysis of Patients With Lung Cancer in the LCS Cohort **eTable 3.** Survival Analysis of Patients With Lung Cancer in the IPN Cohort **eFigure 1.** Percentage of Patients Who Were or Were Not Diagnosed With Lung Cancer in the LCS and IPN Cohorts, Stratified by Lung-RADS Score or Nodule Size **eFigure 2.** Survival Comparisons of Patients in the LCS Cohort Stratified by Lung-RADS Score and the IPN Cohort Stratified by Nodule Size **eFigure 3.** Adjusted Hazard Ratios (AHRs) for LCS and IPN Cohorts by Lung-RADS Category and Nodule Size

Supplement 2.
Data Sharing Statement
