# Supplementary material for: Lung Cancer Diagnosis Rates in Early Detection Programs in the Mississippi Delta
Source: JAMA Netw Open. 2026 Apr 13;9(4):e263171. doi: 10.1001/jamanetworkopen.2026.3171 (PMC13077516; doi:10.1001/jamanetworkopen.2026.3171)
Supplement: Supplement 1. — eTable 1. Cancer Characteristics of DELUGE Patients With Lung Cancer eTable 2. Survival Analysis of Patients With Lung Cancer in the LCS Cohort eTable 3. Survival Analysis of Patients With Lung Cancer in the IPN Cohort eFigure 1. Percentage of Patients Who Were or Were Not Diagnosed With Lung Cancer in the LCS and IPN Cohorts, Stratified by Lung-RADS Score or Nodule Size eFigure 2. Survival Comparisons of Patients in the LCS Cohort Stratified by Lung-RADS Score and the IPN Cohort Stratified by Nodule Size eFigure 3. Adjusted Hazard Ratios (AHRs) for LCS and IPN Cohorts by Lung-RADS Category and Nodule Size [file jamanetwopen-e263171-s001.pdf]

Supplemental Online Content

Liao W, Tye S, Goss J, Fehnel C, Ray M, Osarogiagbon RU. Lung cancer diagnosis rates in early detection programs in the Mississippi Delta. *JAMA Netw Open*. 2026;9(3):e263171. doi:10.1001/jamanetworkopen.2026.3171

eTable 1. Cancer Characteristics of DELUGE Patients With Lung Cancer

eTable 2. Survival Analysis of Patients With Lung Cancer in the LCS Cohort

eTable 3. Survival Analysis of Patients With Lung Cancer in the IPN Cohort

eFigure 1. Percentage of Patients Who Were or Were Not Diagnosed With Lung Cancer in the LCS and IPN Cohorts, Stratified by Lung-RADS Score or Nodule Size

eFigure 2. Survival Comparisons of Patients in the LCS Cohort Stratified by Lung-RADS Score and the IPN Cohort Stratified by Nodule Size

eFigure 3. Adjusted Hazard Ratios (aHRs) for LCS and IPN Cohorts by Lung-RADS Category and Nodule Size

This supplemental material has been provided by the authors to give readers additional information about their work.

© 2026 Liao W et al. *JAMA Network Open*.

eTable 1. Cancer Characteristics of DELUGE Patients With Lung Cancer		
**Cancer characteristics**	**LCS**<br>**N = 401**	**IPN**<br>**N = 1139**
**Histology (n, %)**		
Not reported	1 (0.25)	3 (0.26)
Adenocarcinoma	178 (44.39)	612 (53.73)
Squamous	129 (32.17)	254 (22.3)
Adenosquamous	1 (0.25)	12 (1.05)
Large cell	12 (2.99)	28 (2.46)
Small cell	44 (10.97)	115 (10.1)
Other	36 (8.98)	115 (10.1)
**Primary tumor size (cm)**		
Not reported	18 (4.49)	99 (8.69)
Mean (SD)	2.26 (1.58)	2.67 (3.07)
Median (Q1 - Q3)	1.9 (1.3 - 2.7)	2.1 (1.5 - 3)
(Min - Max)	(0.3 - 13.5)	(0.5 - 80)
**Clinical stage (n, %)**		
Not reported	12 (2.99)	71 (6.23)
Stage I	251 (62.59)	595 (52.24)
Stage II	23 (5.74)	60 (5.27)
Stage III	55 (13.72)	177 (15.54)
Stage IV	60 (14.96)	236 (20.72)
**Treatment (n, %)**		
**Surgery alone**	147 (36.66)	332 (29.15)
**Chemo alone**	40 (9.98)	128 (11.24)
**Radiation alone**	69 (17.21)	187 (16.42)
**No treatment**	17 (4.24)	60 (5.27)
**Hospice**	5 (1.25)	24 (2.11)
Chemo + Rad	61 (15.21)	156 (13.7)
Surgery + Chemo	27 (6.73)	65 (5.71)
Surgery + Rad	1 (0.25)	6 (0.53)
Surgery + Rad + Chemo	5 (1.25)	10 (0.88)
SBRT	62 (15.46)	117 (10.27)
**Follow-up from diagnosis (days)**		
Mean (SD)	745 (659)	879 (807)
Median (Q1 - Q3)	569 (240 - 1022)	647 (248 - 1279)

© 2026 Liao W et al. *JAMA Network Open*.

(Min - Max)	(0 - 3228)	(0 - 3379)
**Crude overall survival (95% CI)**		
Median (Q1 - Q3)	NA (NA - NA)	4.09 (3.42 - 5.3)
**1-year**		
Aggregate	82 (78, 86)	73 (70, 76)
**3-year**		
Aggregate	69 (64, 75)	56 (53, 59)
**5-year**		
Aggregate	63 (56, 71)	46 (43, 50)

DELUGE = Detecting Early Lung Cancer; LCS = Lung Cancer Screening; IPN, Incidental Pulmonary Nodule; LC = lung cancer; SD = standard deviation; Q1 = first quartile; Q3 = third quartile; Min = minimum; Max = maximum; Chemo = chemotherapy; Rad = radiation; SBRT = stereotactic body radiation therapy. To compare characteristics, we used chi-square tests (Fisher's exact test if cell counts expectedly small) and Cramer's *V* for effect sizes for categorical variables; Wilcoxon rank-sum tests and Cohen's *f* for effect sizes for continuous variables. Survival differences between cohorts were assessed using the log-rank test.

© 2026 Liao W et al. *JAMA Network Open*.

eTable 2. Survival Analysis of Patients With Lung Cancer in the LCS Cohort						
Survival	LCS					
	RADS1-2<br>N = 114	RADS3<br>N = 54	RADS4A<br>N = 77	RADS4B<br>N = 79	RADS4X<br>N = 68	*P* value
**Duration of follow-up from enrollment to diagnosis (days)**						
Mean (SD)<br>Median (Q1 - Q3)<br>(Min - Max)	1047 (671)<br>875 (509 - 1463)<br>(19 - 3585)	1128 (705)<br>995 (555 - 1561)<br>(31 - 3316)	1006 (742)<br>813 (415 - 1442)<br>(7 - 3206)	761 (709)<br>574 (98 - 1271)<br>(7 - 2 991)	591 (648)<br>172 (37 - 898)<br>(9 - 3205)	<.001
**Crude overall survival (95% CI)**						
**1-year**						
Aggregate	80 (72, 89)	80 (69, 94)	83 (74, 93)	90 (83, 97)	80 (71, 90)	.90
Stage I	92 (85, 99)	92 (82, 100)	85 (76, 96)	96 (90, 100)	95 (88, 100)	.68
Stage II	100 (100, 100)	50 (13, 100)	80 (52, 100)	88 (67, 100)	100 (100, 100)	.16
Stage III	69 (50, 96)	86 (63, 100)	83 (58, 100)	86 (63, 100)	61 (39, 95)	.81
Stage IV	35 (17, 74)	46 (20, 100)	62 (32, 100)	54 (26, 100)	50 (28, 88)	.13
**3-year**						
Aggregate	65 (55, 78)	69 (55, 87)	70 (58, 84)	71 (59, 84)	73 (63, 86)	.90
Stage I	75 (62, 90)	87 (74, 100)	78 (66, 91)	78 (65, 93)	84 (72, 98)	.68
Stage II	100 (100, 100)	0 (NA, NA)	80 (52, 100)	52 (25, 100)	100 (100, 100)	.16
Stage III	58 (35, 94)	43 (10, 100)	50 (22, 100)	86 (63, 100)	61 (39, 95)	.81
Stage IV	23 (8, 70)	46 (20, 100)	31 (7, 100)	36 (12, 100)	50 (28, 88)	.13
**5-year**						
Aggregate	60 (48, 76)	69 (55, 87)	64 (51, 82)	58 (44, 77)	69 (57, 84)	.90
Stage I	75 (62, 90)	87 (74, 100)	78 (66, 91)	65 (49, 88)	78 (62, 97)	.68
Stage II	100 (100, 100)	0 (NA, NA)	80 (52, 100)	52 (25, 100)	100 (100, 100)	.16
Stage III	58 (35, 94)	43 (10, 100)	50 (22, 100)	86 (63, 100)	61 (39, 95)	.81
Stage IV	0 (NA, NA)	46 (20, 100)	0 (NA, NA)	0 (NA, NA)	50 (28, 88)	.13

N indicates the total number of participants diagnosed with lung cancer in a category. LCS = Lung Cancer Screening; RADS = Lung Imaging and Reporting and Data System; SD = standard deviation, Q1 = first quartile; q3 = third quartile; Min = minimum; Max = maximum; CI = confidence interval. For comparisons of crude overall survival, *P* values were derived from log-rank tests.

© 2026 Liao W et al. *JAMA Network Open*.

eTable 3. Survival Analysis of Patients With Lung Cancer in the IPN Cohort					
**Survival**	**IPN**				
	**(0, 6) mm N = 89**	**[6,15] mm N = 549**	**(15, 20] mm N = 233**	**(20,30] mm N = 268**	***P* value**
**Duration of follow-up from enrollment to diagnosis (days)**					
Mean (SD)	1279 (887)	1341 (921)	1203 (939)	1028 (909)	<.001
Median (Q1-Q3)	1079 (541 - 1873)	1155 (554 - 2044)	1014 (398 - 1946)	797 (227 - 1656)	
(Min - Max)	(0 - 7514)	(0 - 3576)	(0 - 3388)	(0 - 3537)	
**Crude overall survival (95% CI)**					
**1-year**					
Aggregate	71 (61, 82)	73 (70, 78)	77 (72, 83)	70 (64, 76)	.19
Stage I	93 (84, 100)	92 (88, 95)	91 (86, 96)	85 (79, 92)	.41
Stage II	100 (100, 100)	79 (65, 95)	88 (67, 100)	65 (46, 92)	.17
Stage III	70 (50, 96)	53 (43, 66)	69 (55, 85)	57 (44, 74)	.02
Stage IV	44 (28, 69)	41 (33, 52)	40 (27, 60)	42 (29, 59)	.94
**3-year**					
Aggregate	54 (42, 69)	55 (50, 60)	63 (56, 70)	52 (45, 59)	.19
Stage I	74 (56, 98)	74 (68, 80)	75 (68, 84)	68 (60, 77)	.41
Stage II	50 (13, 100)	49 (32, 75)	88 (67, 100)	32 (16, 66)	.17
Stage III	55 (34, 87)	30 (20, 45)	52 (37, 72)	40 (27, 58)	.02
Stage IV	35 (20, 61)	26 (19, 37)	28 (16, 48)	26 (16, 45)	.94
**5-year**					
Aggregate	50 (37, 67)	47 (42, 53)	50 (43, 59)	42 (35, 50)	.19
Stage I	63 (42, 96)	64 (57, 71)	59 (50, 70)	55 (45, 66)	.41
Stage II	50 (13, 100)	42 (25, 70)	88 (67, 100)	32 (16, 66)	.17
Stage III	55 (34, 87)	20 (11, 37)	47 (31, 69)	28 (17, 49)	.02
Stage IV	35 (20, 61)	26 (19, 37)	19 (7, 49)	20 (9, 43)	.94

N indicates the total number of participants diagnosed with lung cancer in a category. IPN = Incidental Pulmonary Nodule; mm = millimeter; SD = standard deviation, Q1 = first quartile; q3 = third quartile; Min = minimum; Max = maximum; CI = confidence interval). For comparisons of crude overall survival, *P* values were derived from log-rank tests.

© 2026 Liao W et al. *JAMA Network Open*.

eFigure Legends.

eFigure 1. Percentage of Patients Who Were or Were Not Diagnosed With Lung Cancer in the LCS and IPN Cohorts, Stratified by Lung-RADS Score or Nodule Size. LCS, Lung Cancer Screening; IPN, Incidental Pulmonary Nodule; Lung-RADS, Lung Imaging and Reporting Data System.

eFigure 2. Survival Comparisons of Patients in the LCS Cohort Stratified by Lung-RADS Score and the IPN Cohort Stratified by Nodule Size. Survival comparisons of patients in (A) the LCS cohort stratified by Lung-RADS score (1, 2, 3, 4A, 4B, and 4X); and (B) the IPN cohort stratified by nodule size (<6, ≥6–15, >15–20, and >20–30 mm). Survival curves were compared using log-rank tests: (A) χ^2 = 1.60, df = 4, *P* = .90; (B) χ^2 = 4.77, df = 3, *P* = .19. LCS, Lung Cancer Screening; IPN, Incidental Pulmonary Nodule; Lung-RADS, Lung Imaging and Reporting Data System.

eFigure 3. Adjusted Hazard Ratios (aHRs) for LCS and IPN Cohorts by Lung-RADS Category and Nodule Size. With Lung-RADS 1-2 as reference, the aHR for lung cancer diagnosis was 3.21 (95% CI, 2.26–4.54), 3.87 (3.27–4.58), 22.86 (16.35–31.96), and 56.83 (40.03–80.67) for Lung-RADS 3, 4A, 4B & 4X in the LCS cohort (all *P*<.001). With nodules 6–15 mm as reference, aHR was 0.23 (0.18–0.30), 3.87 (3.27–4.58) and 5.80 (4.92–6.85) among patients in the IPN cohort with nodules <6 mm, ≥15–20 mm, and >20–30 mm, respectively (all *P*<.001). All *P* values are from Wald tests in multivariable Cox proportional hazards models adjusted for age, sex, race, insurance, rurality, comorbidities, personal history of non-lung

cancer, and family history of cancer. LCS, Lung Cancer Screening; IPN, Incidental Pulmonary Nodule; Lung-RADS, Lung Imaging and Reporting Data System.

© 2026 Liao W et al. *JAMA Network Open*.

eFigure 1.

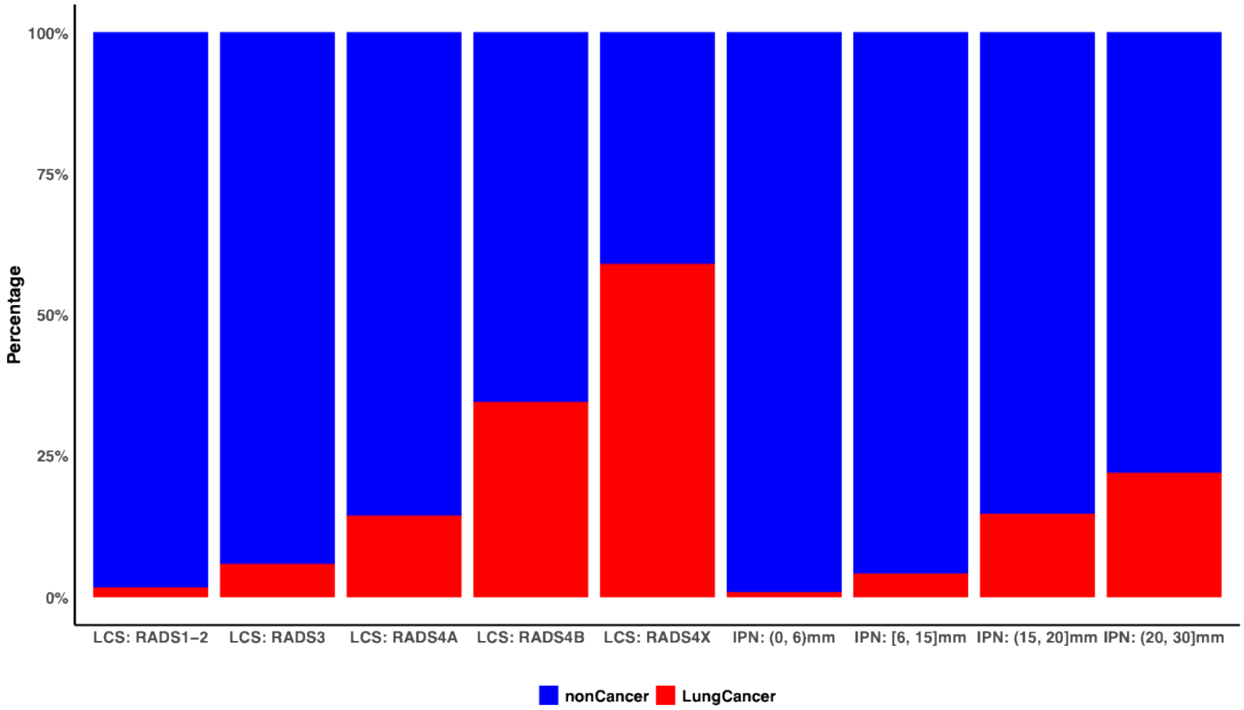

© 2026 Liao W et al. *JAMA Network Open*.

eFigure 2A.

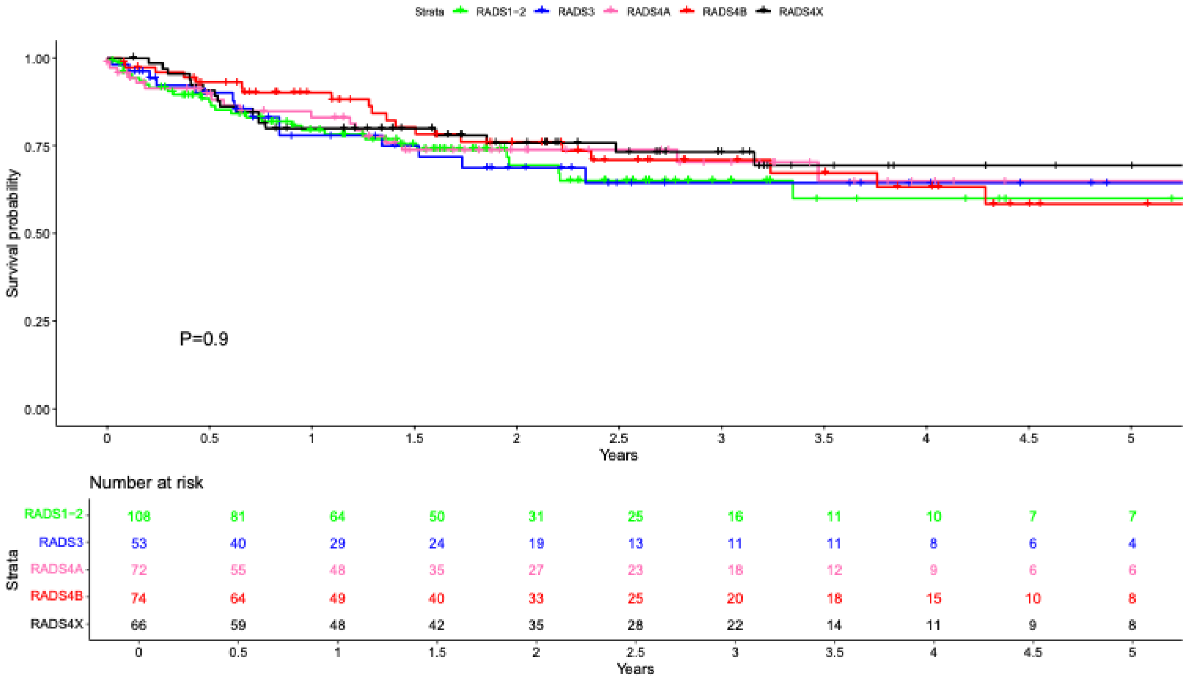

© 2026 Liao W et al. *JAMA Network Open*.

eFigure 2B.

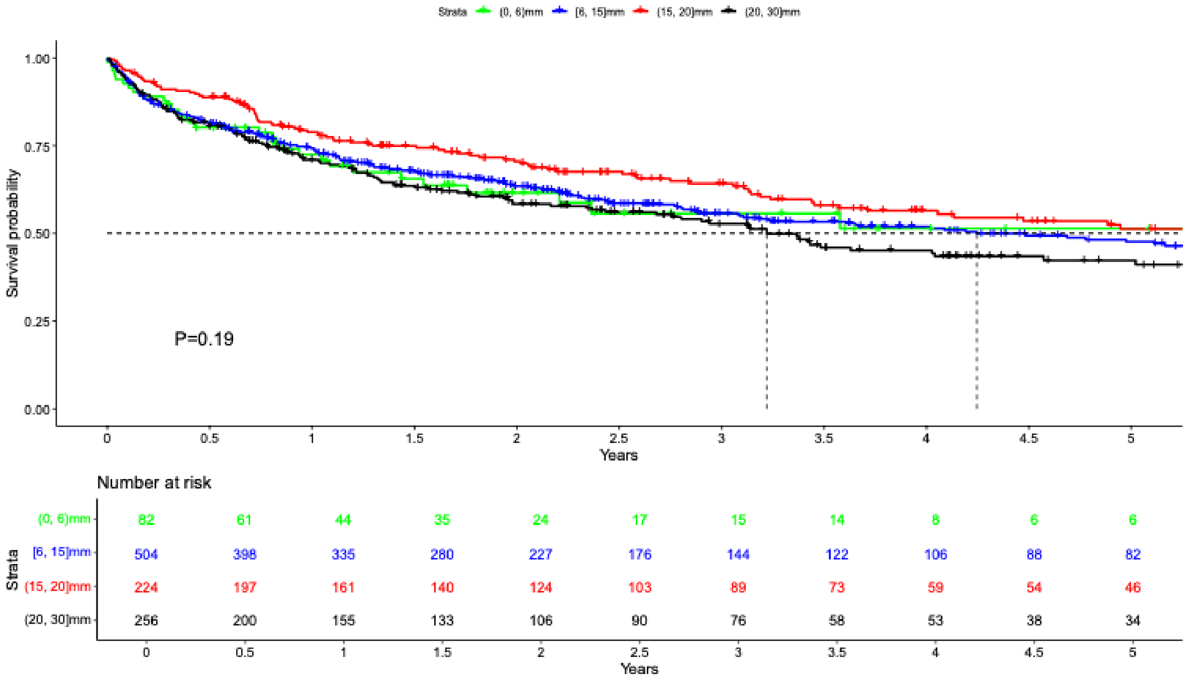

© 2026 Liao W et al. *JAMA Network Open*.

eFigure 3.

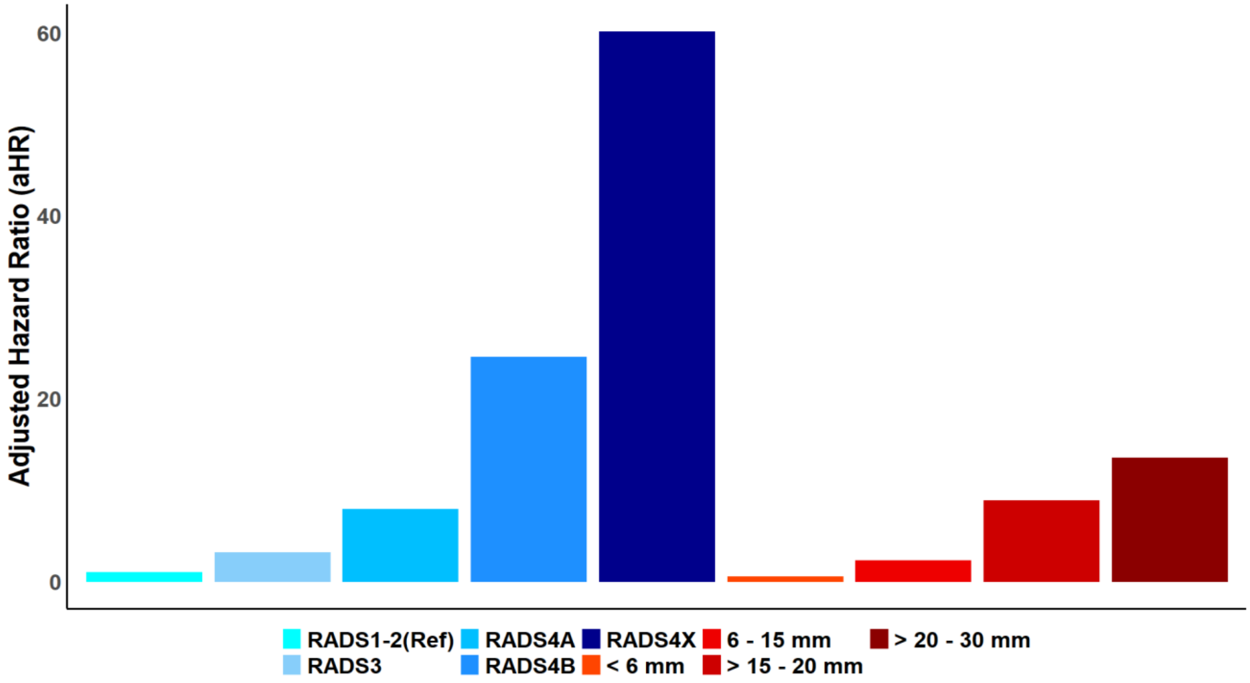

© 2026 Liao W et al. *JAMA Network Open*.
