# Supplementary material for: Lung Cancer Diagnosis Rates in Early Detection Programs in the Mississippi Delta
Source: JAMA Netw Open. 2026 Apr 13;9(4):e263171. doi: 10.1001/jamanetworkopen.2026.3171 (PMC13077516; doi:10.1001/jamanetworkopen.2026.3171)
Supplement: Supplement 2. — Data Sharing Statement [file jamanetwopen-e263171-s002.pdf]

Data Sharing Statement

Data
Data available: No

Additional Information
Explanation for why data not available: Data used in this study were derived from a limited dataset, within the Baptist IRB approved REDCap database, titled ‘Detecting Early Lung Cancer (DELUGE) in the Mississippi Delta cohort.’ Summary level data may be shared upon request if approved through the Baptist legal and regulatory channels.
